# Supplementary material for: Light-driven lattice soft microrobot with multimodal locomotion
Source: Nat Commun. 2025 Aug 28;16:8059. doi: 10.1038/s41467-025-62676-z (PMC12394689; doi:10.1038/s41467-025-62676-z)
Supplement: Supplementary file 1 — Supplementary Information [file 41467_2025_62676_MOESM1_ESM.pdf]

Supplementary Information for

Light-driven Lattice Soft Microrobot with Multimodal Locomotion

Mingduo Zhang[1†], Yuncheng Liu[1†], Chunsan Deng[1], Xuhao Fan[1], Zexu Zhang[1], Shaoxi Shi[1], Fayu Chen[1],Huace Hu[1], Leimin Deng[1,2], Lige Liu[3], Tao Sun[3,4], Hui Gao[1,2], Wei Xiong[1,2*]

[1]Wuhan National Laboratory for Optoelectronics, School of Optical and Electronic Information, Huazhong University of Science and Technology, Wuhan, China.

[2]Optics Valley Laboratory; Wuhan, Hubei, China.

[3]State Key Laboratory of High End Heavy Load Robots, Foshan, 528300, China.

[4]Artificial Intelligence Research Center, Midea Group, Shanghai, 201702, China.

[*]Corresponding author. Email: weixiong@hust.edu.cn

The file includes:

Supplementary Note 1

Supplementary Fig. S1 to Fig. S13

Supplementary Table 1

Other Supplementary Information for this manuscript includes the following:

Movies S1 to S9

Supplementary Note

Note S1. Discussion of the relationship between movement speed and laser scanning speed

The movement displacement can be obtained,

$$D = L_{step} \cdot N_{scanning} \tag{S1}$$

where D is the motion displacement of the LSMR, L_{step} is the single peristalsis step displacement, and $N_{scanning}$ is the number of laser scans. The single peristalsis step displacement depends on the displacement of the contraction process minus the displacement of the recovery process shown in Fig. 3 of the main text and is calculated as,

$$L_{step} = L_b \left[f_s(t) - f_d(t) \right] \tag{S2}$$

where L_b is the length of the deformation region at the end of the robot. f_s and f_d describe the shrinking radio of time in the shrinking and swelling processes, respectively. t is the time for the laser to pass through the deformation region. The number of laser scans depends on the laser scanning speed, scanning time, and the length of the single scan, which can be calculated as,

$$N_{scanning} = \frac{v_s t_s}{L_s} \tag{S3}$$

where v_s is the laser scanning speed, t_s is the laser scanning time, and L_s is the laser scanning distance within a single cycle.

Inserting subsequently Equation(S2) and Equation(S3) into Equation(S1) a expression for D can be obtained:

$$D = L_b \left[f_s(t) - f_d(t) \right] \frac{v_s t_s}{L_s} \tag{S4}$$

Meanwhile, the movement distance of LSMR can be expressed as,

$$D = v_l t_s \tag{S5}$$

where v_l is the movement speed of the LSMR. The time of laser to pass through the deformation region can be calculated as,

$$t = \frac{d}{v_s} \tag{S6}$$

Inserting subsequently Equation(S5) and Equation(S6) into Equation(S4) a expression for v_l can be obtained:

$$v_l = L_b \left[f_s\left(\frac{d}{v_s}\right) - f_d\left(\frac{d}{v_s}\right) \right] \frac{v_s}{L_s} \tag{S7}$$

The variation of shrinking radio with time during shrinking and swelling can be obtained by fitting the data shown in Fig. 2e in Results. The shrinking process shrinking radio can be expressed as,

$$f_s = A_2 + \frac{A_1 - A_2}{1 + e^{t - x_0/dx}} \quad \text{(S8)}$$

where A_1=0.00975, A_2 = 0.2027, x_0 = 0.33745, dx = 0.1106. Here, A1 represents the theoretical shrinkage ratio at the initial state (baseline level), which is very close to zero, consistent with the physical expectation that no significant shrinkage occurs at the beginning. A2 corresponds to the final stable shrinkage plateau value, matching the experimentally observed terminal shrinkage ratio (0.2027). x0 denotes the center time of the shrinkage transition, that is, the inflection point where the shrinkage accelerates and then slows down, indicating a very fast hydrogel response. dx is the scale factor describing the steepness of the transition; the fitted value of 0.11 s suggests a relatively rapid change from fast shrinkage to the stable state. The swelling process shrinking radio can be expressed as,

$$f_d = y_0 + A\left(\frac{frac}{1 + e^{t - x_{01}/k_1}} + \frac{1 - frac}{1 + e^{t - x_{02}/k_2}}\right) \quad \text{(S9)}$$

where y_0=-0.0136, A=0.21649, $frac$=0.56217, x_{01}=0.74557, x_{02}=2.19965, k_1=-0.36545, k_2=-0.09528. Here, y0 represents a small initial baseline offset, close to zero, which indicates a correction to the starting point, possibly due to baseline measurement error or a slight shift in the fitting. A represents the overall swelling amplitude (i.e., the edge length change relative to the fully swollen state), reflecting the maximum deformation as the hydrogel transitions from the contracted state to the fully swollen state. The final plateau value is approximately A + y0 (≈ 0.21649 - 0.0136 ≈ 0.2027), consistent with the stable swelling condition. frac indicates the contribution of the fast swelling component, accounting for 56.2% of the total swelling amplitude, suggesting that the majority of swelling is dominated by the fast stage. x01 is the center time of the fast swelling phase, representing the characteristic time scale of this rapid transition. x02 is the center time of the slow swelling phase, indicating the inflection point of the slower swelling component. k1 reflects the steepness of the fast stage; the fitted value of approximately -0.37 s indicates that the major swelling occurs within the first ~1.5 seconds. The negative sign is a conventional feature of the Boltzmann formulation (indicating direction of change). k2 corresponds to the steepness of the slow stage; although the fitted value (about -0.095 s) implies a relatively sharp transition, the large x02 value means the change actually occurs more gradually in the later phase. All fitting parameters were determined through iterative fitting in Origin using the Levenberg-Marquardt algorithm, which combines gradient descent and Gauss-Newton methods.

To determine the parameters of Equation S8 and S9, we performed an experimental fit of the shrinkage and swelling kinetics under laser stimulation (Fig. 2e). The resulting fitted curves and extracted coefficients are presented in Supplementary Fig. S7a.

Inserting subsequently Equation(S6), Equation(S8) and Equation(S9) into Equation(S7) a expression for v_l can be obtained:

$$v_l = L_b\left[\left(A_2 + \frac{A_1 - A_2}{1 + e^{d/v_s - x_0/dx}}\right) - \left(y_0 + A\left(\frac{frac}{1 + e^{d/v_s - x_{01}/k_1}} + \frac{1 - frac}{1 + e^{d/v_s - x_{02}/k_2}}\right)\right)\right]\frac{v_s}{L_s} \quad \text{(S10)}$$

Supplementary Figure

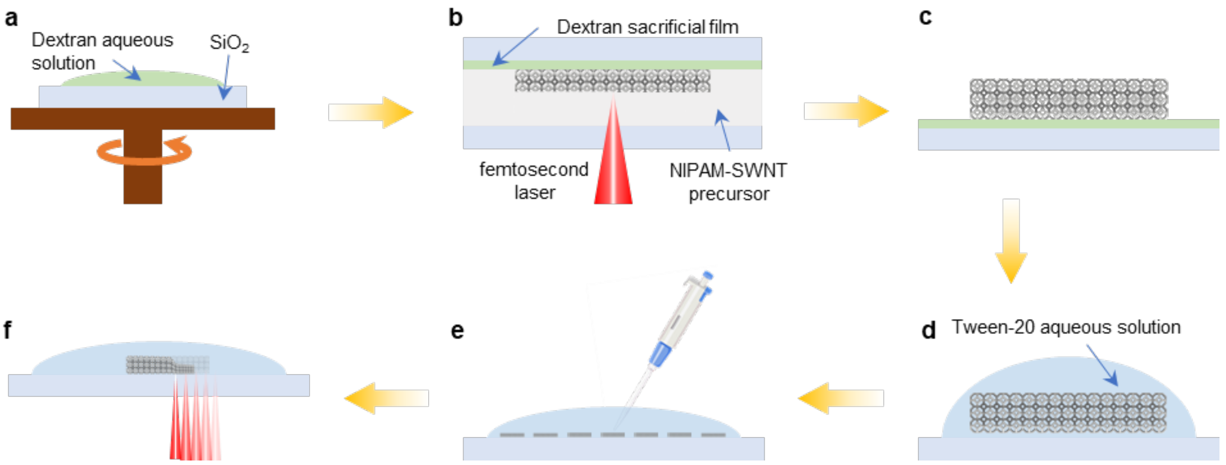

Fig. S1. Schematic illustration of the manufacturing process of the lattice soft microrobots. a Spin-coating sacrificial layer. **b** The process of dirct laser writing. **c** Dissolve unpolymerized precursors. **d** Dissolve the sacrificial layer to release the LSMR from the substrate. **e** Transform the LSMR to application scenarios by a pipette, the image of the pipette was provided by figdraw.com. **f** Light driven the LSMR movement.

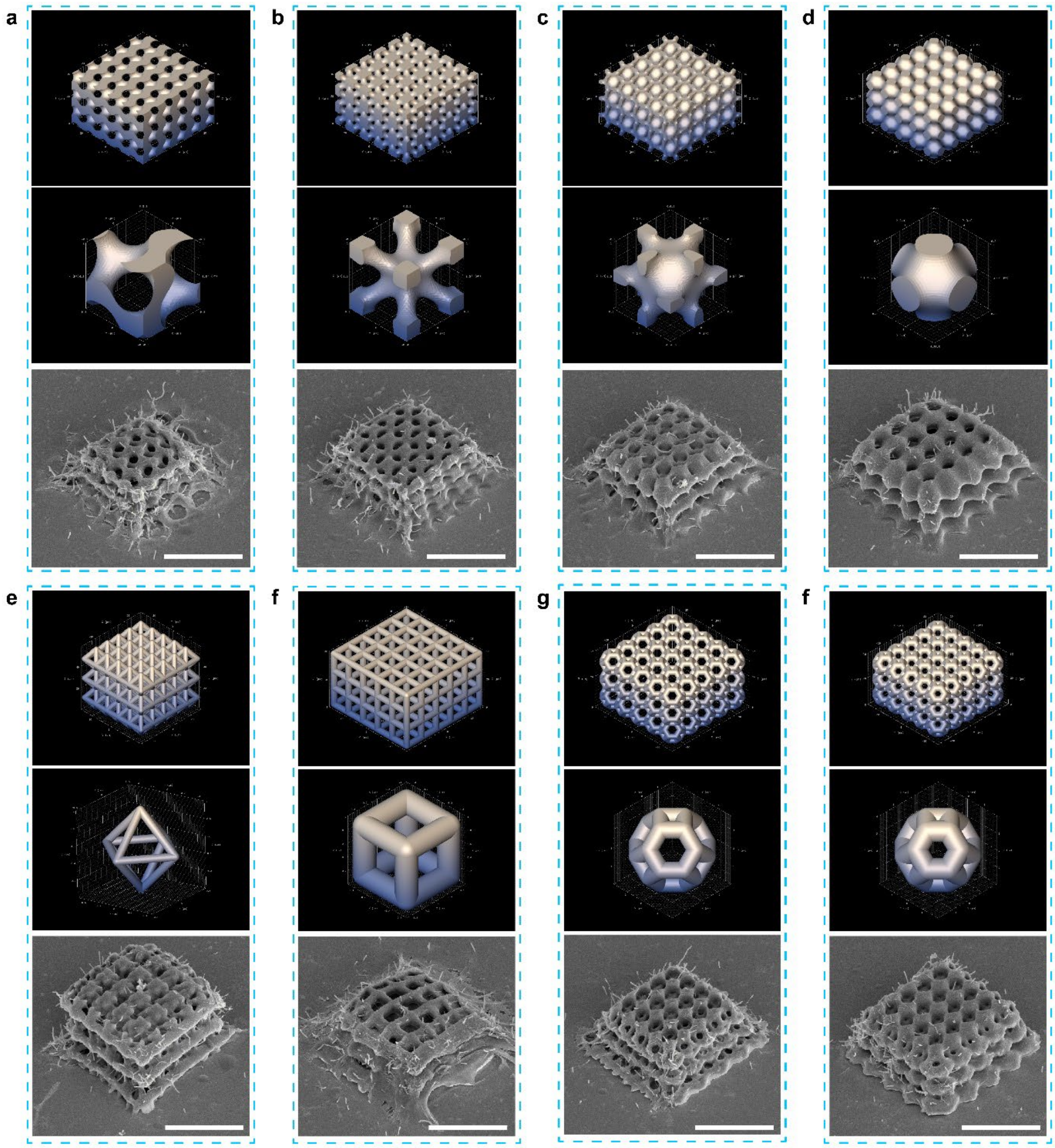

Fig. S2. Different types of lattice architecture. Triple-period minimum surface type lattice, including: **a** gyroid, **b** I-WP, **c** neovius, **d** primitive. Truss type lattice, including: **e** octahedral lattice, **f** square lattice, **g** truncated octahedra, **h** truncated octahedra. All scales bar: 30 μm.

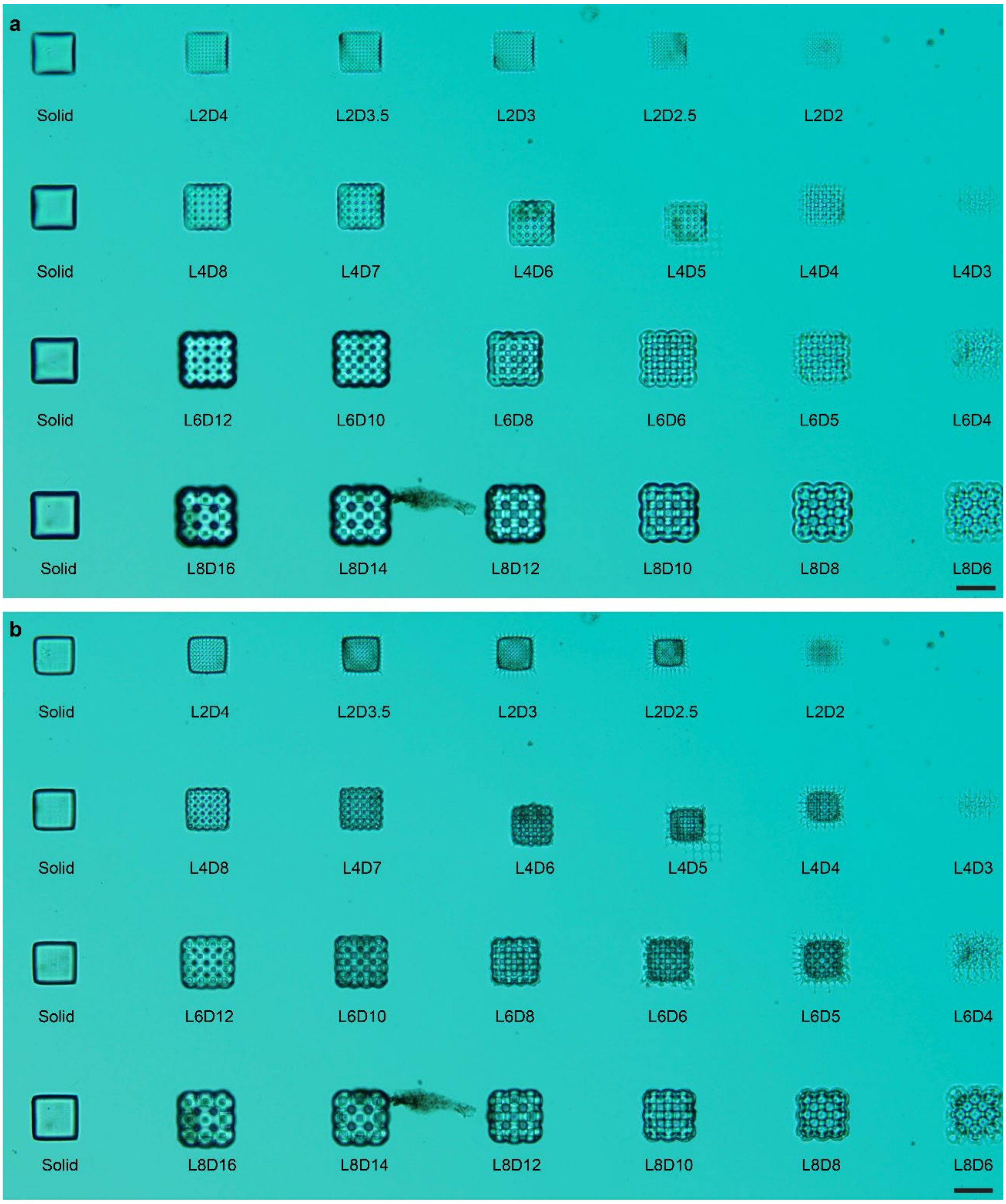

Fig. S3 Optical microscopy images of lattice structure in the swollen and shrunken states. a Optical microscopy images of solid structures and lattice structures of multiple parameters in the swelling state. Scale bar: 50 μm. **b** Optical microscopy images of solid structures and lattice structures of multiple parameters in the shrinking state. Scale bar: 50 μm.

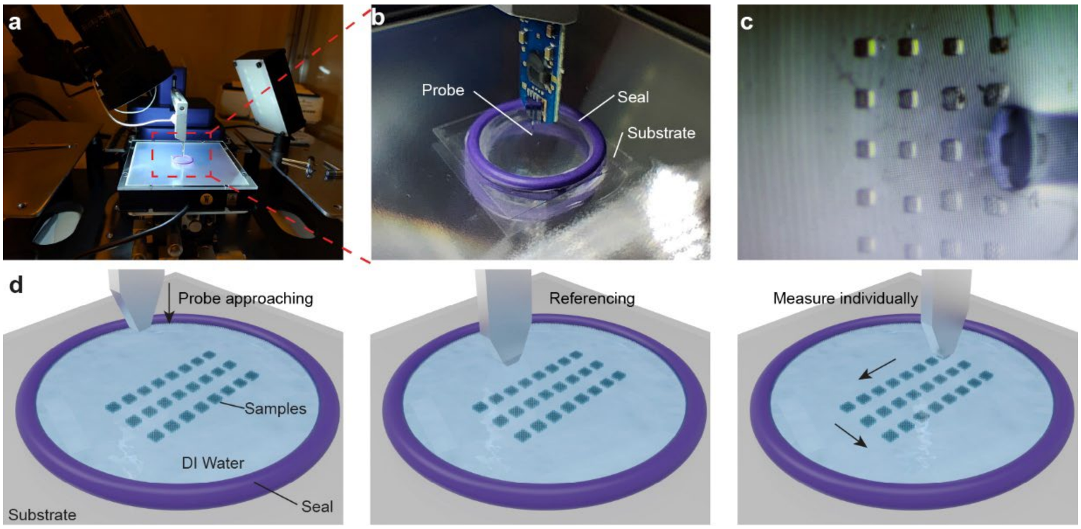

Fig. S4. Schematic diagram of the PNIPAM-SWNT hydrogel mechanical property test setup and process. **(a)** Mechanical test setup. On the left side is the micro-imaging system for real-time observation of the testing process. On the right side and below is the illumination system. The displacement stage at the back drives the fixed arm as well as the probe for stiffness testing. The system captures the displacement of the probe and the force exerted by the probe in real time. **(b)** Water immersion test device. An O-ring seal was pasted on the coverslip surface with UV-curable resin. Deionized water was dripped inside the seal to keep the water surface level. The probe penetrated deep inside the deionized water to measure the PNIPAM-SWNT hydrogel block in the dissolved state. **(c)** Photograph of microscopic imaging of the measurement process. **(d)** Schematic of the measurement process. The probe was firstly penetrated deep into the water by visualization. Then the stiffness value brought by the substrate is measured in the blank, and then the measured value is input into the system for correction. Finally, each hydrogel block is measured in turn to obtain the corresponding displacement-force data.

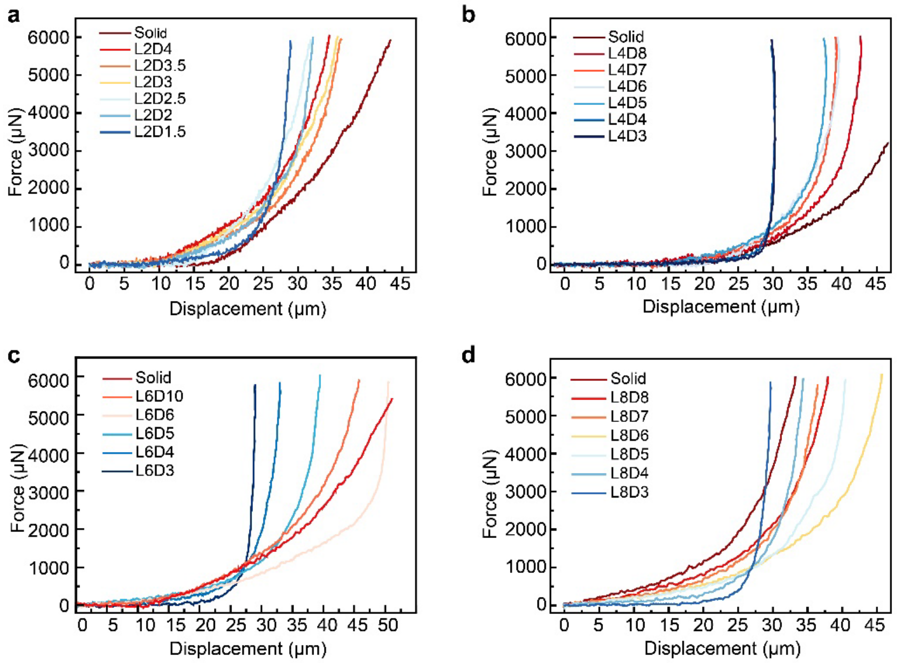

Fig. S5. Mechanical properties testing of lattice structures. **a** Force-displacement curves for lattice and solid structures with the rod length of 2 µm. **b** Force-displacement curves for lattice and solid structures with the rod length of 4 µm. **c** Force-displacement curves for lattice and solid structures with the rod length of 6 µm. **d** Force-displacement curves for lattice and solid structures with the rod length of 8 µm.

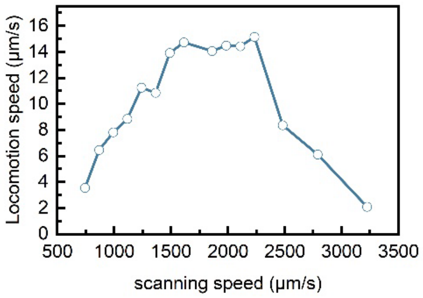

Fig. S6 Change of microrobot speed as a function of laser scan speeds.

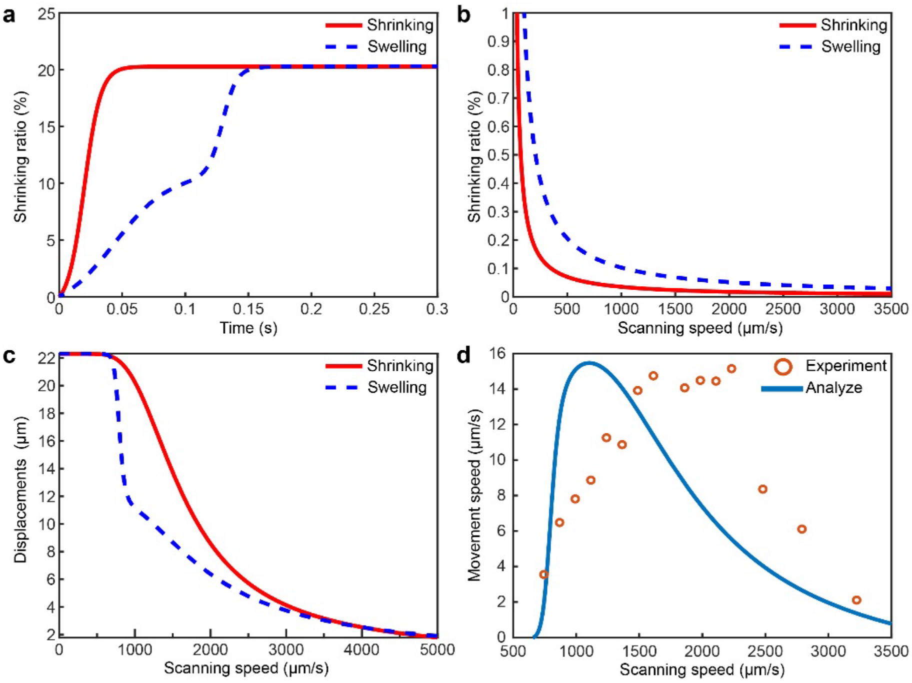

Fig. S7 Correlation of LSMR movement speed and laser scanning speed. a Shrinking ratio of lattice structure at shrinking process and swelling process. **b** LSMR shrinking ratio at different laser scanning speeds. The LSMR body length is 110 μm, the laser heat-affected zone diameter is 28 μm, and the laser single scan distance is 600 μm. **c** End displacement of LSMR at different scanning speeds. **d** LSMR movement speed at different scanning speeds.

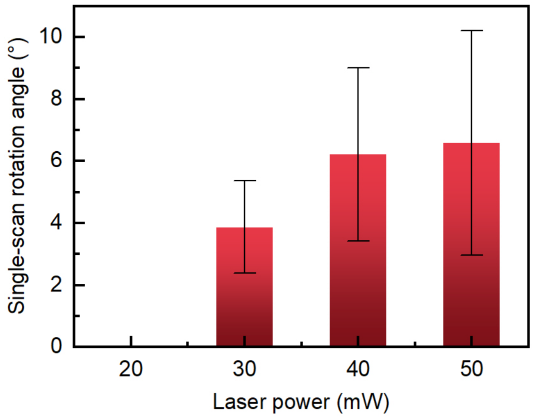

Fig. S8. The mean angular precision of LSMR under different laser power levels. Angular precision is defined as the angle of rotation of the LSMR per laser scan rotation.

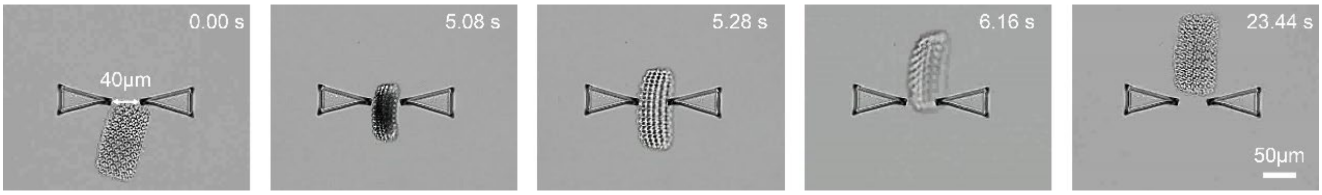

Fig. S9. The LSMR with a body width of 60 μm crossed a 40 μm slit, flipped sideways, and floated during travel.

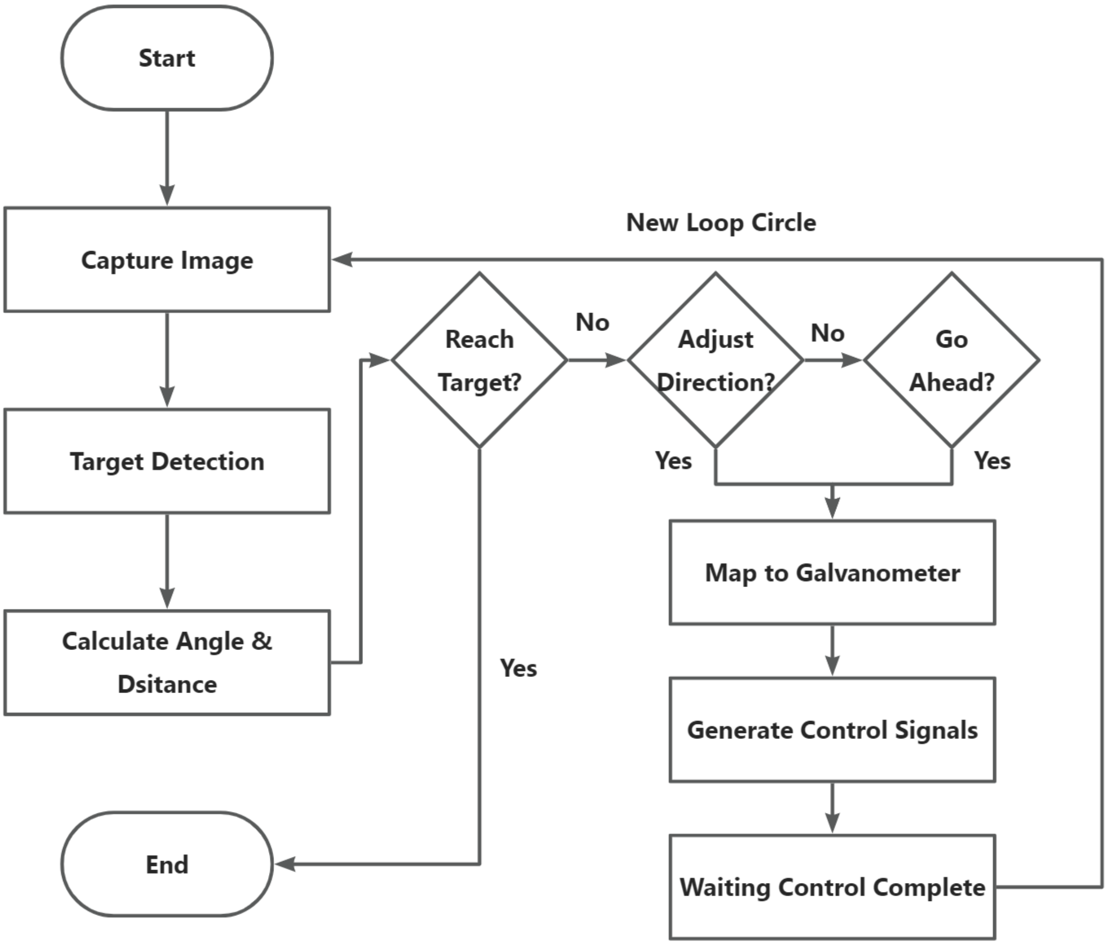

Fig. S10. The program flowchart of the closed-loop control system

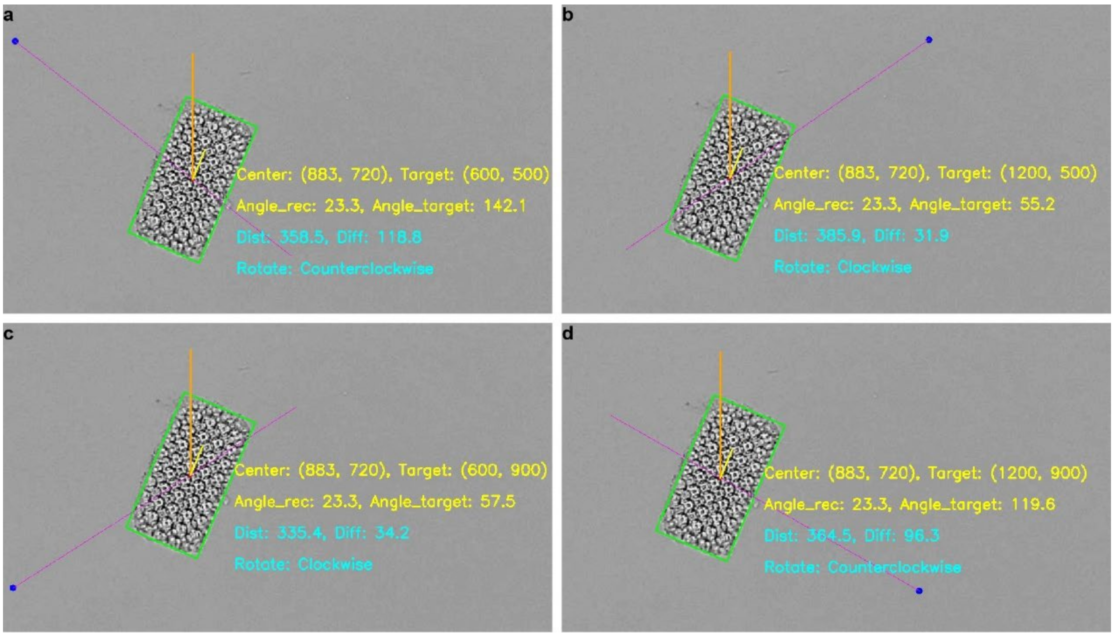

Fig. S11 detailed method to adjust direction in the closed loop control scheme. The green rectangles represent the outlines of the LSMRs recognized by python-based OpenCV. The blue points represent the artificially set target points. The orange line represents the artificially set 0-degree line used for reference. The yellow line represents the direction of the long side of the rectangle. The purple line represents the line between the target point and the center of the rectangle. Angle_rec represents the angle between the long side of the rectangle and the 0 degree line at this point in time. Angle_targle represents the angle between the line between the center of the rectangle and the target point and the 0 degree line. Dist represents the distance between the center of the rectangle and the target point, in pixels. Diff represents the angle between the direction of the long side of the rectangle and the target point. a b c and d correspond to different cases where the target point is in the center of the rectangle in each of the four directions.

First, we use Python-OpenCV to extract the contour of the LSMR and fit it with a minimum bounding rectangle, as shown by the green rectangular box. We then obtain the center point of the rectangle and the angle between its longer side and the vertical axis. We define the center of the fitted rectangle as the origin, with the upward vertical direction as 0°, as shown by the orange line. Since the LSMR is a symmetric structure, we do not distinguish between its head and tail, allowing us to unify its orientation within the range of 0° to 180°. The LSMR angle is determined by rotating clockwise from the 0° reference line until it aligns with the longer side of the rectangle.

Next, we determine the target point angle by constructing a line from the rectangle's center to the target point. The target angle is defined by rotating clockwise from the 0° reference line until it aligns with this constructed line. Similar to the LSMR angle, the target angle is also unified within the 0° to 180° range. The angle difference between the target point and the LSMR is then computed. If the angle difference is less than 90°, the laser is controlled to scan clockwise, as illustrated in Fig. S11 b and c. If the angle difference is greater than 90°, the laser is controlled to scan counterclockwise, as shown in Fig. S11 a and d. This method ensures precise directional adjustments in the closed-loop control scheme.

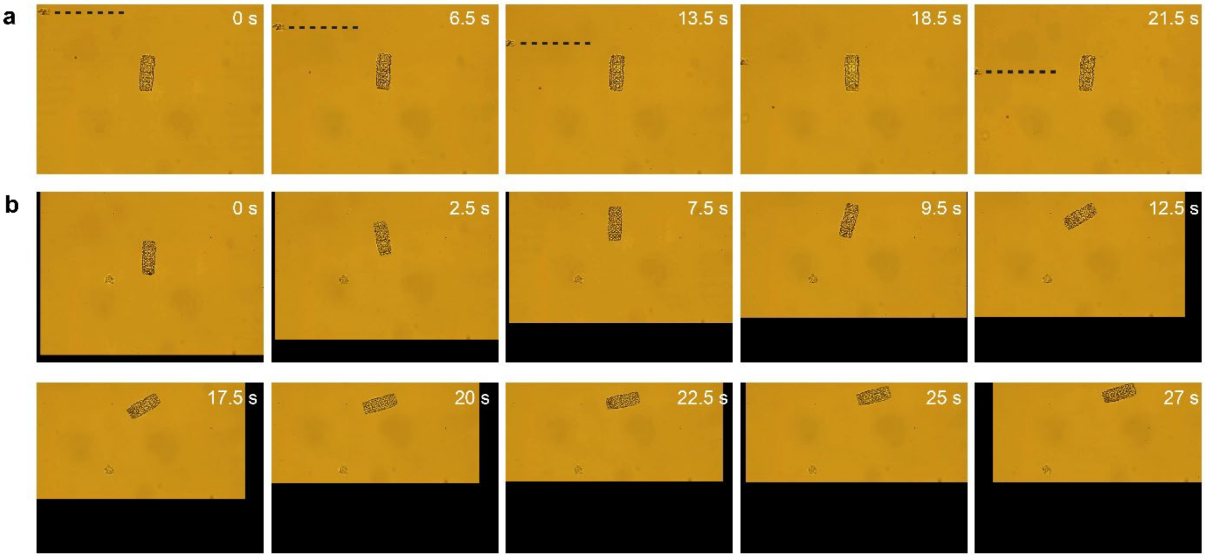

Fig. S12. Continuous skipping based on the thermophoretic effect. a Decomposed images of the linear continuous jump superimposed image. **b** Decomposed images of the superimposed image.

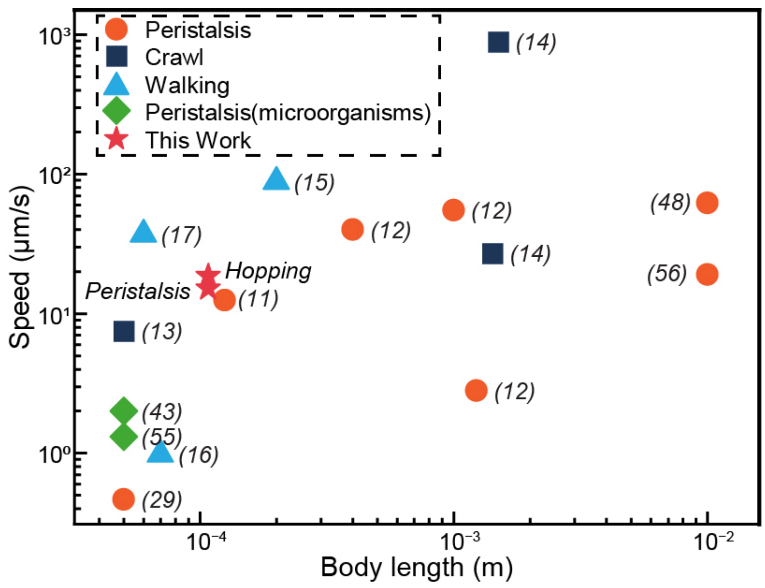

Fig. S13 Summary of reported robots plotted as the ratio of locomotion speed (μm/s) to body length as a function of length. The figure includes soft microrobots based on creeping, crawling, walking, and natural microbial peristalsis.

Supplementary Table

Table S1. Design parameters of lattice structures shown in Fig. S3

Design parameters (L*x*D*y*)	Block length design value (µm)	Block height design value (µm)	Corresponding solid structure volume(mm^3)	Lattice structure volume(mm^3)	Relative density
L2D1	57.569	34.941	1.158×10^{-4}	1.94×10^{-5}	16.75%
L2D1.5	58.069	35.441	1.195×10^{-4}	3.87×10^{-5}	32.38%
L2D2	58.569	35.941	1.233×10^{-4}	5.97×10^{-5}	48.42%
L2D2.5	59.045	36.433	1.270×10^{-4}	8.00×10^{-5}	62.98%
L2D3	59.569	36.941	1.311×10^{-4}	9.93×10^{-5}	75.76%
L2D3.5	60.12	37.5	1.355×10^{-4}	1.13×10^{-4}	83.25%
L2D4	60.02	38	1.369×10^{-4}	1.23×10^{-4}	89.85%
Cube	60	60	2.160×10^{-4}		100.00%
L4D2	58.67	36.02	1.240×10^{-4}	2.10×10^{-5}	16.97%
L4D3	59.56	36.94	1.310×10^{-4}	4.20×10^{-5}	32.02%
L4D4	60.6	37.98	1.395×10^{-4}	6.54×10^{-5}	46.90%
L4D5	61.6	38.96	1.478×10^{-4}	8.86×10^{-5}	59.96%
L4D6	62.58	39.96	1.565×10^{-4}	1.11×10^{-4}	70.74%
L4D7	63.58	40.96	1.656×10^{-4}	1.29×10^{-4}	77.92%
L4D8	64.58	41.96	1.750×10^{-4}	1.44×10^{-4}	82.27%
Cube	60	60	2.160×10^{-4}	-	100.00%
L6D3	70.97	53.99	2.719×10^{-4}	4.60×10^{-5}	16.92%
L6D4	71.86	54.89	2.834×10^{-4}	7.44×10^{-5}	26.25%
L6D5	72.93	55.97	2.977×10^{-4}	1.10×10^{-4}	36.83%
L6D6	73.92	56.96	3.112×10^{-4}	1.44×10^{-4}	46.41%
L6D8	75.91	58.94	3.396×10^{-4}	2.13×10^{-4}	62.72%
L6D10	77.9	60.94	3.698×10^{-4}	2.75×10^{-4}	74.46%
L6D12	79.9	62.93	4.017×10^{-4}	3.24×10^{-4}	80.53%
Cube	60	60	2.160×10^{-4}	-	100.00%
L8D4	71.865	49.233	2.543×10^{-4}	4.23×10^{-5}	16.64%
L8D6	73.944	51.292	2.805×10^{-4}	8.74×10^{-5}	31.16%
L8D8	75.9	53.27	3.069×10^{-4}	1.37×10^{-4}	44.64%
L8D10	77.902	55.284	3.355×10^{-4}	1.90×10^{-4}	56.48%
L8D12	79.882	57.255	3.654×10^{-4}	2.40×10^{-4}	65.61%
L8D14	81.9	59.277	3.976×10^{-4}	2.85×10^{-4}	71.68%
L8D16	83.882	61.255	4.310×10^{-4}	3.25×10^{-4}	75.41%
Cube	60	60	2.160×10^{-4}	-	100.00%
