# Supplementary material for: Light-driven lattice soft microrobot with multimodal locomotion
Source: Nat Commun. 2025 Aug 28;16:8059. doi: 10.1038/s41467-025-62676-z (PMC12394689; doi:10.1038/s41467-025-62676-z)
Supplement: Supplementary file 2 — Description of Additional Supplementary Files [file 41467_2025_62676_MOESM2_ESM.pdf]

Description of Additional Supplementary Files

Supplementary Movie 1: Light response deformation of lattice structure with different relative densities.

Supplementary Movie 2: Ordered peristalsis deformation of microrobots under sequential laser scanning.

Supplementary Movie 3: Comparison of energy conversion rates between lattice and solid structures.

Supplementary Movie 4: Continuous linear peristalsis and in-situ rotation of microrobot.

Supplementary Movie 5: Microbot squeeze through narrow slit.

Supplementary Movie 6: Manual control of the micro-robot's “H” path movement.

Supplementary Movie 7: Programmed motion manipulated by the closed-loop feedback control system.

Supplementary Movie 8: Thermophoresis-based straight-line and right-turn continuous hopping.

Supplementary Movie 9: Continuous hopping to maneuver the maze based on thermophoretic.
